# Investigation into the effect of hepatitis B virus on apoliprotein A1 expression and its mechanism

**DOI:** 10.1186/1476-511X-13-130

**Published:** 2014-08-13

**Authors:** Weichao Jiang, Lei Zheng, Qianqian Yang, Zhouying Huang, Xiaobei Wang

**Affiliations:** Department of Clinical Laboratory, Union Hospital, Tongji Medical College, Huazhong University of Science and Technology, Wuhan, Hubei 430022 P. R. China

**Keywords:** Apolipoprotein A1, Hepatitis B virus, High-density lipoprotein cholesterol

## Abstract

**Background:**

Hepatitis B virus (HBV) infection poses a serious threat to human health, with China being one of the highly affected countries. However, the pathogenesis of chronic hepatitis B (CHB) is still unclear. Apolipoprotein A1 (ApoA1) which represents the major protein component of high-density lipoprotein is normally secreted by hepatocytes. When hepatocytes are infected with HBV may lead to the disruption of ApoA1 secretion. In this study, we investigated the effect of HBV on ApoA1 expression and preliminarily explored its molecular mechanism of regulation for revealing the pathogenesis of CHB.

**Methods:**

The expression of mRNA and protein of ApoA1 in Human HepG2 hepatoblastoma cells and subline HepG2.2.15 cells were performed by reverse transcription-polymerase chain reaction (RT-PCR) and Western-blot. The serum ApoA1, by the immune turbidimetric test, and high-density lipoprotein cholesterol (HDL-C) in CHB patients and healthy controls, based on the enzymatic method, were measured with autobiochemical analyzer. The statistical difference was analyzed by SPSS 13.0. HBV infectious clone, pHBV1.3, and ApoA1 gene promoter were co-transfected into HepG2, and the luciferase activity was determined. The changes of ApoA1 mRNA and protein expression were detected by RT-PCR and Western-blot method, after HepG2 cells were transfected with pHBV1.3.

**Results:**

The expression of ApoA1 mRNA and protein in HepG2.2.15 were lower than those in HepG2, and when compared with healthy controls, serum levels of ApoA1 and HDL-C in CHB patients were lower (P < 0.05). pHBV1.3 in HepG2 cells restrained the activity of ApoA1 promoter, mRNA and protein expression.

**Conclusions:**

HBV could inhibit the expression of ApoA1 in vitro and in vivo.

## Background

Hepatitis B virus (HBV) infection has a devastating effect on human health. According to statistics, there are about 3 billion people with HBV infection in the whole world. China is one of the highly affected countries in the world; approximately 120 million people are carrying HBV, and about a quarter of HBV patients develop chronic hepatitis disease, including chronic hepatitis, liver fibrosis, liver cirrhosis and liver cancer [1]. So far, the pathogenesis of CHB remains unclear. In a previous study, we explored the pathogenesis of CHB by screening the differential expression genes between the hepatocellular carcinoma cell line HepG2 and HepG2.2.15 cell lines (integrated the HBV genome) by gene chip and found that the expression of ApoA1 in HepG2.2.15 was significantly decreased [2]. Apoliprotein A1 (ApoA1) which represents the major protein component of high-density lipoprotein is normally secreted by hepatocytes. When hepatocytes are infected with HBV may lead to the disruption of ApoA1 secretion. An inverse correlation between HBV and ApoA1 was found in two hepatoma cell lines [3]. Plasma ApoA1 was decreased in chronic hepatitis B patients [4]. The current research is mainly to study the effect of HBV on ApoA1 expression, and preliminarily explore its molecular mechanism of regulation as a basis for revealing the pathogenesis of chronic hepatitis.

## Materials and methods

### Experiment design

Our experiment is mainly divided into two parts. First, plasma ApoA1 was investigated in CHB patients and healthy controls to explore the relationship between HBV and ApoA1, considering that ApoA1 is normally secreted by hepatocytes. Then a cell-cuture experiment was performed to illuminate whether ApoA1 expression could be influenced by HBV.

### Study subjects

The study recruited 118 clinically diagnosed chronic hepatitis B (CHB) patients from Union Hospital, Tongji Medical College, Huazhong University of Science and Technology, including 66 males and 52 females. All subjects were Chinese Han ethnic origin and from the Central China. All subjects were given Informed Consent Forms and the study protocols were approved by the Hospital Ethics Committee. The mean age was 46.3 ± 15.4 years. Case selection was consistent with the diagnostic criteria of “viral hepatitis prevention and treatment measure” which were jointly revised by the Infectious and Parasitic diseases branch and Hepatology of the Chinese Medical Association in 2000. The patients who had a history of critical organs disease such as heart, brain and kidney disease or other chronic liver disease were excluded. Another 102 healthy subjects (57 males and 45 females) were selected as controls, the mean age was 43.7 ± 14.5 years. There was no statistical difference between the gender, age and height of CHB patients and healthy controls (*P* > 0.05).

### Materials

Human hepatoma cell lines, HepG2 and HepG2.2.15, were provided by Central Laboratory, Union Hospital, Tongji Medical College, Huazhong University of Science and Technology. HBV infectious clone, pHBV1.3 (Genbank number: U95551), was constructed by our laboratory. Liposomal transfection (lipofectamine 2000) and RNA extraction reagents (TRIzolR) were purchased from Invitrogen Company, and M-MLV reverse transcriptase was purchased from Promega Corporation (America). ApoA1 monoclonal antibody was purchased from Shanghai Gaochuang Chemical Technology Co., Ltd.

### Methods

1. ApoA1 and HDL-C detection: ApoA1, by the immune turbidimetric test, and HDL-C, based on the enzymatic method, were measured by Olympus 5400 automatic biochemical analyzer.
2. Cell culture and transfection:Human HepG2 hepatoblastoma cells and subline HepG2.2.15 cells were cultured in the RPMI-1640 medium containing 10% fetal bovine serum, 100 U/mL penicillin and 100 mg/L streptomycin, in 37°C incubator with 5% volume fraction of CO2. Before transfection, HepG2 cells were seeded in 6-well plates. The transfection method was as follows, different doses of plasmids and lipofectamine 2000 transfection reagents were diluted in 100 μL RPMI-1640 medium without serum and double-antibody, at room temperature for 20 minutes, the prepared transfection solution was added to the medium of HepG2 cells and cultured in the CO2 incubator.
3. RNA isolation and cDNA synthesis: Total RNA was isolated using RNA purification columns (Qiagen). cDNA was synthesized using cDNA Synthesis Kit (Qiagen) with the primer as T7-Oligo (dT) 15(5’-AAACGACGGCCA GTGAATTGTAATACGACTCACTATAGGCGCTTTTTTTTTTTTTTTTV-3’, V might be G, C and A). cRNA was synthesized using T7 RiboMAX Express Large Scale RNA Production System (Promega). cRNA was reverse transcribed with M-MLV reverse transcriptase (Invitrogen). The products of cRNA reverse transcription were labeled with KLENOW enzyme. The final concentrations of dATP, dGTP and dTTP were all 120 μmol/L, the final concentration of dCTP was 60 μmol/L, and the final concentrations of Cy5-dCTP and Cy3-dCTP were both 40 μmol/L.
4. cDNA microarray hybridization and analysis: Purified, labeled cDNA was hybridized in 30 μl of hybridization buffer (25% formamide, 3 × SSC, 0.2% SDS) at 42°C for a whole night. After incubation, the slides were washed and scanned in a LuxScan 10KA scanner (CapitalBio Company, Beijing, China).
5. RT-PCR: This was performed using TRIzolR reagent. Total RNA was extracted from cells and cDNA was synthesized by reverse transcription. ApoA1 gene was amplified by PCR with the forward primer 5'-ATGAAAGCTGCGGTGCTGA-3' and the reverse primer 5'-TCACTGGGTGTTGAGCTTC-3', β-actin was chosen as an internal reference. The final products were analyzed by 1% agarose gel electrophoresis.
6. Determination of luciferase: HepG2 cells were cultured for 48 h after transfection, the supernatant was removed and the cells were washed by PBS. A cell lysis buffer was added to lyse the cells. When the cells were completely lysed, 50 μL cells lysate and 50 μL luciferin substrate were added to it. The optical density was then measured using a Luminometer.
7. Western-blot: HepG2 cells were lysed by ultrasonication, and proteins in the supernatant were extracted after centrifugation. The protein concentration was determined by coomassie brilliant blue G250 method. 30 μg of sample proteins and an equal volume of loading buffer were subjected to 12% SDS-PAGE and transferred onto nitrocellulose (NC) membranes. The membranes were subsequently incubated with ApoA1 monoclonal antibodies (1:2000) for two hours. It was further incubation with horseradish peroxidase conjugated goat anti-rabbit secondary antibody (1:500) for one hour. Visualization was performed by the electrochemical luminescence (ECL) method. Images were digitally acquired using Kodak 4000MM image station and densitometry was conducted using the companion software (Carestream Molecular Imaging, New Heaven, CT).

### Statistical analysis

All data were statistically analyzed by SPSS13.0, and the data were expressed as mean ± standard deviation. The variances were the same (*P* > 0.05). Two-sample t-test was used to evaluate the differences between the ApoA1 and HDL-C levels of CHB patients and controls. *P* value of less than 0.05 was considered statistically significant.

## Results

### The serum ApoA1 and HDL-C levels of patients with HBV infection

**Figure 1:**
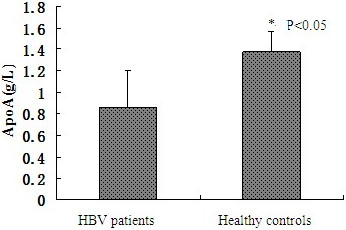
Comparison of serum ApoA1 levels between healthy controls and HBV patients, * ***P*** **< 0.05.**

**Figure 2:**
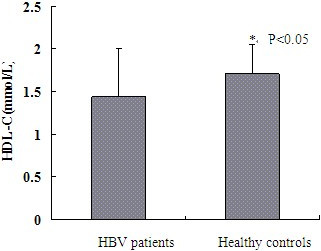
Comparison of serum HDL-C levels between healthy controls and HBV patients, * ***P*** **< 0.05.**

ApoA1 levels in healthy controls (1.37 ± 0.19 g/L) were higher than that in HBV patients (0.86 ± 0.34 g/L, *P* < 0.05; see Figure 1). HDL-C in HBV patients (1.44 ± 0.56 mol/L) was significantly lower than that in healthy controls (1.71 ± 0.34 mmol/L, *P* < 0.05; see Figure 2).

### HBV suppression of ApoA1 mRNA and protein expression

**Figure 3:**
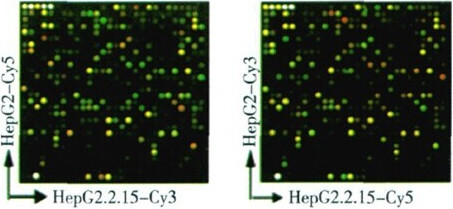
**Exchange images of two kinds of fluorescences of HepG2 vs HepG2.2.15.**

We screened the differential expression genes between HepG2 and HepG2.2.15 cells by gene chip technology. As a result, cy3/cy5 (R/G value), the fluorescence intensity of ApoA1 in HepG2.2.15 and HepG2 cells was 0.0514 which showed that there were significant differences between the expression of ApoA1 in HepG2.2.15 and in HepG2 cells, and the transcription in HepG2.2.15 cells decreased (see Figure 3).

**Figure 4:**
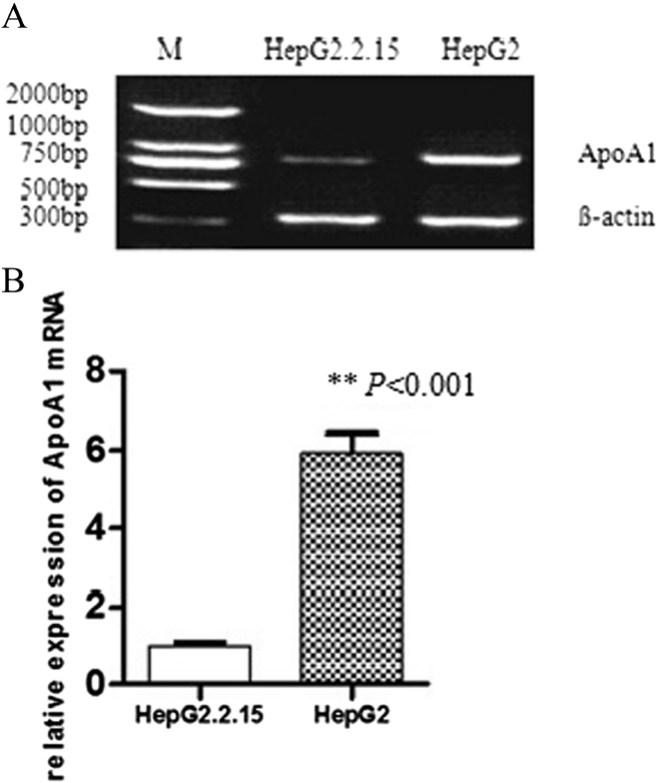
**HBV suppression of ApoA1 mRNA expression.** ApoA1 mRNA expression between HepG2 and HepG2.2.15 cells was measured by RT-PCR. Representative images are shown in **A** and quantification data in **B**. M represents Marker. **P < 0.001.

**Figure 5:**
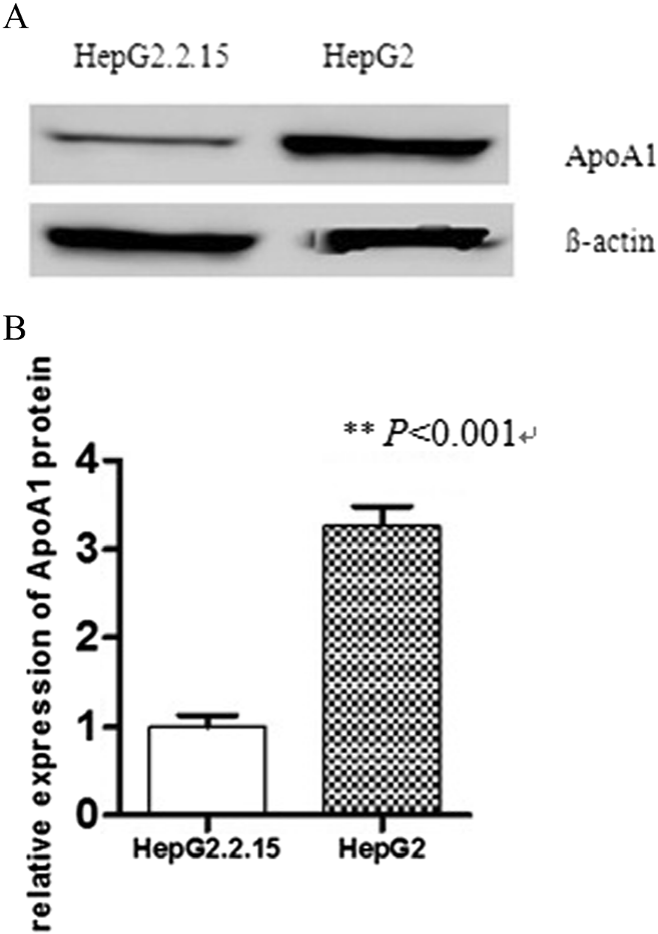
**HBV suppression of ApoA1 protein expression.** ApoA1 protein expression between HepG2 and HepG2.2.15 cells was measured by western-blot. Representative images are shown in **A** and quantification data in **B**. **P < 0.001.

We confirmed the result of gene chip by RT-PCR and Western-blot, the results showed that the expression of ApoA1 mRNA and protein in HepG2 was significantly higher than those in HepG2.2.15 (*P* < 0.001, see Figures 4 and 5).

These results suggested that HBV could downregulate the expression of ApoA1 mRNA and protein in cells.

### HBV inhibition of ApoA1 promoter activity

**Figure 6:**
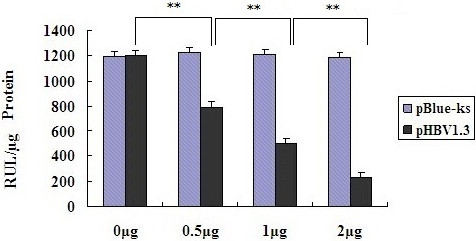
Effect of different doses of pHBV1.3 on the activity of ApoA1 promoter in HepG2 cells, ** ***P*** **< 0.001.**

Different doses of HBV infectious clone pHBV1.3 (0 μg, 0.5 μg, 1 μg, 2 μg) with ApoA1 promoter pApoA1-Luc co-transfected into HepG2 cells, with pBlue-ks as blank control. The results of luciferase activity exhibited that the activity of ApoA1 promoter decreased with the increasing content of pHBV1.3 (0 μg, 0.5 μg, 1 μg, 2 μg), this were 1205.62 ± 43.14RUL/μg, 792.77 ± 37.27RUL/μg, 496.59 ± 26.31RUL/μg and 229.42 ± 16.49RUL/μg respectively (*P* < 0.001, see Figure 6).

### HBV inhibition of the expression of ApoA1 mRNA and protein

**Figure 7:**
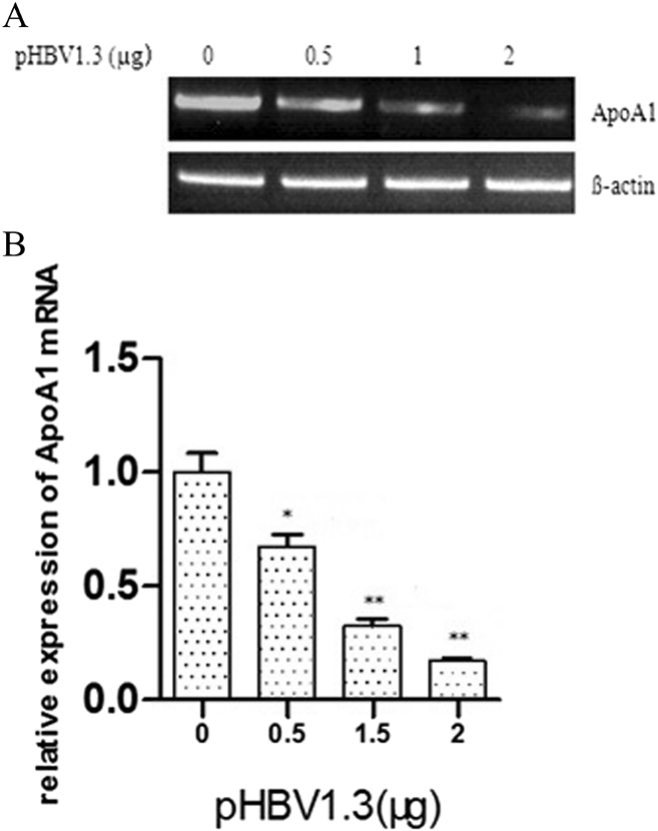
**HBV inhibition of the expression of ApoA1 mRNA.** The expression of ApoA1 mRNA in HepG2 cells inhibited by different doses of pHBV1.3 was measured by RT-PCR. Representative images are shown in **A** and quantification data in **B**. Data were considered significant if P < 0.05(indicated by “*”) and P < 0.001(indicated by “**”).

**Figure 8:**
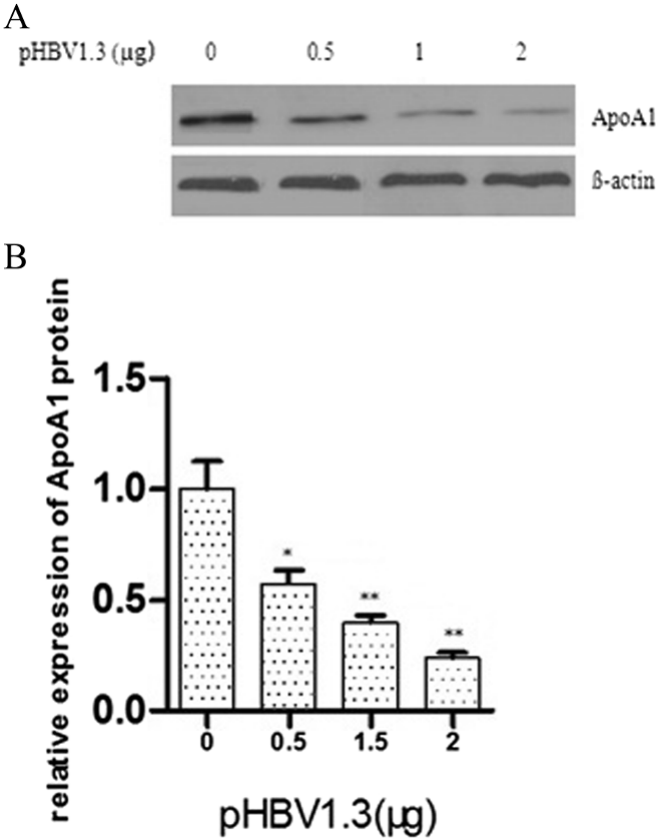
**HBV inhibition of the expression of ApoA1 protein.** The expression of ApoA1 protein in HepG2 cells inhibited by different doses of pHBV1.3 was measured by western-blot. Representative images are shown in **A** and quantification data in **B**. Data were considered significant if P < 0.05(indicated by “*”) and P < 0.001 (indicated by “**”).

Different doses of HBV infectious clone pHBV1.3 (0 μg, 0.5 μg, 1 μg, 2 μg) were transfected into HepG2 cells. The changes of ApoA1 mRNA and protein expression were detected by RT-PCR and Western-blot. As a result, transfection with increasing content of pHBV1.3, the expression of ApoA1 mRNA and protein continued to reduce, in a dose-dependent effect (see Figures 7 and 8).

## Discussion

At present, it is generally considered that liver cells damage is not directly associated with CHB pathogenesis, but caused by inflammatory reaction and immune response of the body after HBV infection [5]. Studies showed that HBV infection could regulate the changes of numerous protein expression, including interleukins 27 (IL-27), IL-29, IL-8 and cyclooxygenase (COX-2), etc. [2, 6].

HepG2.2.15 cell line is a kind of hepatoma cell line stably transfected with the HBV genome, and can express viral RNA and protein, synthetise and secrete the complete virus-like particles. To further explore the pathogenesis of CHB, differential expression genes in HepG2.2.15 and HepG2 cells were screened by gene chip, a series of differentially expressed proteins were found, such as ApoA1. A previous research results indicated that HBV inhibited the synthesis and secretion of ApoB by the regulation of microsomal triglyceride transfer protein (MTP) expression [7].

Gene chip technology has the characteristics of high flux and high sensitivity, but there are certain false positive and false negative at the same time. We confirmed the result of gene chip by RT-PCR and Western-blot. Many studies have shown that the level of circulating ApoA1 correlates with CHB and CHB related disease [3, 8–10]. Our results suggested HBV inhibited the expression of ApoA1 mRNA and protein. Furthermore we detected the serum ApoA1 level in CHB patients and healthy controls. The decrease of serum ApoA1 levels indicated that HBV could inhibit the expression of ApoA1 mRNA and protein in vitro. ApoA1 is the carrier of free cholesterol in vivo, and the major structural protein of HDL-C. The serological level of HDL-C in these two groups was further tested. The serum levels of HDL-C in CHB patients were lower than that in the healthy controls, which demonstrated that HBV affected the serum HDL-C levels by inhibiting the expression of ApoA1.

To explore the molecular mechanism of ApoA1 expression regulated by HBV, we achieved ApoA1 promoter (−474 + 7) by PCR amplification, cloned it to pGL3-basic vector with luciferase reporter gene and constructed ApoA1 promoter luciferase reporter gene pApoA1-Luc [11]. The regulation of HBV on ApoA1 promoter was measured with fluorescent reporter gene system, and the change of ApoA1 mRNA and protein expression was detected by RT-PCR and Western-blot. ApoA1 expression in promoter, mRNA and protein levels, were inhibited by HBV, in a dose-dependent effect. All these indicated that HBV was likely to down regulate the activity of ApoA1 promoter to inhibit ApoA1 mRNA and protein expression. However, the signal pathway of the regulation of HBV on ApoA1 expression and its exact mechanism remained unknown.

A number of studies had shown that viral infections could cause the disorder of lipoprotein metabolism in vivo, such as ApoA1, ApoB, HDL-C, low density lipoprotein cholesterol (LDL-C), total triglyceride (TG) and other apolipoproteins, and lipoprotein levels in serum were reduced after heptatitis C virus (HCV) infection [12–14]. In other studies, an inverse correlation between HBV and ApoA1 was found in two hepatoma cell lines [3] and plasma ApoA1 was decreased in chronic hepatitis B patients [4]. Consistent with these findings, in our research, we investigated the effect of HBV on ApoA1 expression, and found out that HBV inhibited the synthesis and secretion of ApoA1 and HDL-C at the same time. HBV infection could cause acute or chronic inflammation of the body and studies have confirmed the anti-inflammatory effect of HDL-C [15]. HBV might suppress the synthesis and secretion of HDL-C by inhibiting ApoA1 expression, and then promote the inflammatory response. Information from previous research of HBV effect on ApoB expression and this current research make it clear that HBV infection can also lead to the disorders of lipid metabolism. However, whether HBV infection as well as HCV infection is able to cause liver steatosis remains unclear.

## Conclusions

In conclusion, this study explored the HBV regulation for ApoA1 from the perspectives of clinical and cellular levels which revealed the pathogenesis of CHB and provided the theoretical basis for clinical diagnosis and treatment of CHB.
